# The mysteries of type 2 diabetes in developing countries

**DOI:** 10.2471/BLT.16.030416

**Published:** 2016-04-01

## Abstract

More research is needed in low- and middle-income countries where the epidemic of type 2 diabetes is on the rise. KM Venkat Narayan talks to Fiona Fleck.

Q: How did you become interested in type 2 diabetes research?

A: When I was a physician and public health person working largely on cardiovascular diseases (CVD) in the early 1990s, a leading cause of death in Scotland at the time, I was invited to join a study of people with type 2 diabetes. This study included a work group to develop standards of care and I got to attend the Cambridge Diabetes Epidemiology Seminar. I met Peter Bennett, who was leading the Pima Indian Study at the National Institutes for Health in the United States of America. He invited me to join his team for two years. It was a difficult decision. My wife and I had to leave tenured jobs and move to Phoenix, Arizona. But those two years have extended to 24 years. Spending time with the Pima Indians and studying the disease and the suffering it was causing was a defining experience for me. I sensed that what was happening in the Pima community could happen to the rest of the world.

Q: And it did happen. As a cause of death, diabetes jumped from number 15 in 1990 to number nine in 2010. Which countries now have a high burden of type 2 diabetes and which are tackling this disease in the most effective way?

A: Countries in the Middle East have a very high prevalence. The countries with the largest number of people with type 2 diabetes are China, India and the United States of America (USA). A major concern today is the increasing numbers of people with type 2 diabetes – not just in China and India – but also in other middle-income and low-income countries. The greatest success in the last 10 to 15 years has been in reducing deaths and complications in people with diabetes, such as heart attacks, stroke, amputations and kidney failure, particularly in Finland, the USA and other high-income countries. High-income countries now have effective tools for the control of blood glucose, blood pressure, lipids and screening for early complications and have done useful research to find ways to implement good quality of care.

“I sensed that what was happening in the Pima community could happen to the rest of the world.”

Q: So more people are living with type 2 diabetes than two decades ago?

A: Yes, more people are surviving longer with the disease, but that means there are more and more people with costly complications. For example, diabetes is the leading contributor to the rise in inflation-adjusted health-care costs in the United States.

Q: If it’s so expensive to manage the disease and its complications, why isn’t more done to prevent it?

A: In the last 20 years several large randomized controlled trials in people at high risk for developing diabetes showed that aggressive life-style interventions (24 weeks of structured counselling, individually or in groups to modify diet and activity) can reduce progression to diabetes by 30–60%. That’s huge. The challenge is to implement that approach across national health systems and, even if implemented, the overall impact on prevalence would be modest, as it would only target people at high risk. Effective type 2 diabetes prevention must target people much earlier, but we still don’t know how to do this.

Q: How can we prevent diabetes across whole populations at an earlier stage?

A: There is a huge knowledge gap at two levels. One, some countries [see news feature on previous page] have introduced taxation on sugar and soft drinks, so people are buying less and shifting consumption to other drinks. But we don’t know which drinks they are shifting to or, importantly, whether this measure is helping to reduce obesity. Two, many countries subsidize the wrong foods, such as refined grains and highly processed foods, and while they recommend five portions of fruit and vegetables a day, the global supply of fruit and vegetables is currently unable to provide this. That shortfall in fruit and vegetables is even greater in developing countries. We need to know how best to incentivize the industrial and agricultural sectors to produce more healthy food at affordable prices. We also need to get to know how best to motivate people to engage in more physical activity and to make their diets healthier.

Q: Much of your recent research has been in low- and middle-income countries, why?

A: Information from high-income countries does not necessarily apply to low- and middle-income countries. In south-east Asia and sub-Saharan Africa many thin people are developing type 2 diabetes. So there may be other important factors for the disease apart from being overweight. We have good data on how to treat diabetes and prevent it in high-risk groups, but we need to seek more knowledge on how to do this in developing countries. There is a huge dearth of prospective epidemiological data in low- and middle-income countries and a lack of intervention studies in these settings, and we need to invest in understanding the biology across the populations that are most affected. Why are so many thin people developing type 2 diabetes in low- and middle-income countries, does this suggest a different phenotype? We need to take the research and the epidemiology to where the epidemic is.

Q: What should low- and middle-income countries be doing to tackle their diabetes epidemics?

A: They should deliver good quality care for people with diabetes and they should identify people at high risk of developing diabetes and implement proven life-style interventions. These are the two immediate priorities. If we can reduce the rates of diabetes complications in high-income countries, why not in low- and middle-income countries? The big question is: can these countries afford to scale-up treatment and prevention? We can learn from the experience of other fields. For example, you can capitalize on: task-shifting to non-physician health-care professionals, low-cost medications, technology and telemedicine. In the vaccine and HIV research fields, the costs of interventions and technology have been reduced by shifting research hubs to low- and middle-income countries. We also need to collectively study the effectiveness of the policies that work and get them implemented. We are not going to win the war on diabetes without more research and better data systems in the countries where many people have diabetes and where the diabetes epidemic is growing fast.

Q: How can good quality care be delivered in low- and middle-income countries?

A: We need to find ways of delivering noncommunicable disease (NCD) prevention and care, regardless of the socioeconomic constraints. India has a fantastic infrastructure for tuberculosis through the DOTS programme. A lot of people with tuberculosis also have diabetes, so why don’t we integrate prevention and care into the tuberculosis clinic network or through primary care? In Africa we can do the same with HIV clinics. Just because a country lacks health resources does not mean good quality programmes cannot be implemented. We are talking about task-shifting to help to achieve this. There are many community health workers working in maternal child health who can be deployed for NCD care also, and patient groups have to be empowered.

“We need to take the research and the epidemiology to where the epidemic is.”

*Q: What contribution does WHO’s* Global action plan for the prevention and control of NCDs 2013-2020 *have the potential to make?*

A: WHO has enormous power as a convener and whatever WHO says has a lot of credibility across the world, especially in low- and middle-income countries. There is extremely strong evidence for the effectiveness of better treatment and we should push harder for good quality diabetes care and management and access to essential medicines for diabetes. Developing countries need better monitoring of quality of care, better governance, better health financing models and more standards that governments can use. It’s tempting to argue for large social and policy measures, but they need to be evidence-based. We need a process of trial and error, where we implement national policies but also evaluate them so that we can learn as we go and avoid mistakes. WHO could argue for greater investment in surveillance and research, including basic research, in low- and middle-income countries. 

Q: What contribution can the sustainable development goals (SDGs) make?

A: A lot. The SDGs cannot be achieved without addressing NCDs such as type 2 diabetes. The international community needs to rally for investment in global NCD prevention and control, in the same way as for HIV. It’s a matter of strengthening primary health systems in low- and middle-income countries, where a good package for NCD and cardiovascular disease management costs about US$ 400 per person per year.

Q: Type 2 diabetes is increasingly appearing in young children. How can we stop this?

A: The first cases of young onset were noted in the Pima Indians in the 1970s. At that time it was thought to be a Native American problem, then it appeared in Japanese people and it was thought to be a minority problem, until it occurred in the white population. In the late 1990s at CDC, we found that type 2 diabetes in youth was occurring in all ethnic groups, and that’s how the SEARCH study started. The study has shown that type 2 diabetes is rare in children aged under 10 years, but not uncommon in the 10-to-20 years’ age group. Studies have also shown that young-onset type 2 diabetes is linked to childhood obesity, and to maternal obesity and glucose levels during pregnancy. We published a paper in the *NEJM* in 2014 tracking a nationally representative sample of 7000 kids in the United States aged between five and 14 years. We found that by five years 27.5% of the kids were overweight, including 12.5% who were obese, and that some factors for childhood obesity and diabetes are rooted in pre-school age and maybe in utero. It’s not surprising that the results of large studies of school-based interventions have not been encouraging, because some of the factors driving young-onset type 2 diabetes start in very early life. 

